# Impact of Light and Temperature on the Uptake of Algal Symbionts by Coral Juveniles

**DOI:** 10.1371/journal.pone.0050311

**Published:** 2012-11-21

**Authors:** David Abrego, Bette L. Willis, Madeleine J. H. van Oppen

**Affiliations:** 1 Australian Institute of Marine Science, Townsville, Queensland, Australia; 2 Australian Research Council Centre of Excellence for Coral Reef Studies, and School of Marine and Tropical Biology, James Cook University, Townsville, Queensland, Australia; King Abdullah University of Science and Technology, Saudi Arabia

## Abstract

The effects of temperature and light on the breakdown of the coral-*Symbiodinium* symbiosis are well documented but current understanding of their roles during initial uptake and establishment of symbiosis is limited. In this study, we investigate how temperature and light affect the uptake of the algal symbionts, ITS1 types C1 and D, by juveniles of the broadcast-spawning corals *Acropora tenuis* and *A. millepora*. Elevated temperatures had a strong negative effect on *Symbiodinium* uptake in both coral species, with corals at 31°C showing as little as 8% uptake compared to 87% at 28°C. Juveniles in high light treatments (390 µmol photons m^−2^ s^−1^) had lower cell counts across all temperatures, emphasizing the importance of the light environment during the initial uptake phase. The proportions of the two *Symbiodinium* types taken up, as quantified by a real time PCR assay using clade C- and D-specific primers, were also influenced by temperature, although variation in uptake dynamics between the two coral species indicates a host effect. At 28°C, *A. tenuis* juveniles were dominated by C1 *Symbiodinium,* and while the number of D *Symbiodinium* cells increased at 31°C, they never exceeded the number of C1 cells. In contrast, juveniles of *A. millepora* had approximately equal numbers of C1 and D cells at 28°C, but were dominated by D at 30°C and 31°C. This study highlights the significant role that environmental factors play in the establishment of coral-*Symbiodinium* symbiosis and provides insights into how potentially competing *Symbiodinium* types take up residence in coral juveniles.

## Introduction

Many marine cnidarians form symbioses with dinoflagellates of the genus *Symbiodinium*, gaining significant nutritional resources that underpin the capacity of scleractinian corals to build coral reefs [1], [2]. The increasing frequency and severity of abnormally high seawater temperatures in recent decades have led to both localized and widespread mass coral mortalities and phase shifts on some coral reefs [3], [4], highlighting the need to understand the effects of changing environmental factors on coral-*Symbiodinium* symbioses. Some types of *Symbiodinium* (9 clades are currently recognized, each comprising multiply types [5]) typically confer bleaching resistance, particularly types in clade D, as deduced from changes in symbiont communities during or shortly after bleaching events [6]–[8], [9] but see [10]. In addition, variation in the physiological responses of different *Symbiodinium* types to temperature and light is well documented, both in culture and *in hospite* [10]–[16]. However, the effects of temperature and light on the intial uptake and establishment of symbiosis are poorly known.

In most coral species (>85%), *Symbiodinium* endosymbiosis is established with each new generation by acquisition of *Symbiodinium* from the environment, i.e., horizontal transmission [17]–[19]. A number of studies of *Symbiodinium* uptake at ambient, non-stressful temperatures, either in the field or in controlled experiments, have shown that corals and other cnidarians can be infected by *Symbiodinium* types different from those previously detected in their tissues or those found in parental colonies [12], [20]–[27]. However, there is a gap in current understanding about whether such flexibility is likely to persist and provide options for survival of the coral holobiont during stressful environmental conditions, such as those expected to become more common over the next decades [28]. Only three studies have investigated the onset of symbiosis in broadcast spawning corals at stressful temperatures, and all have focused on *Symbiodinium* uptake in larvae [29]–[31]. The pattern emerging is that uptake of algal symbionts by larvae is lowest at high temperatures (>29°C); moreover, larvae associated with *Symbiodinium* have lower survival rates at high temperatures than those without symbionts. However, it is not known if high temperatures similarly affect establishment of symbiosis in coral juveniles following metamorphosis, an ecologically important stage for *Symbiodinium* uptake, given evidence of order of magnitude greater abundances of *Symbiodinium* in reef sediments compared to the water column [32].

In corals with horizontal symbiont transmission, more than one type of *Symbiodinium*, including non-homologous types, may be taken up over a relatively short period of time at the larval or primary polyp stage. We hypothesize that intrinsic physiological differences between *Symbiodinium* types may confer competitive advantages to some types under different thermal regimes, thus the endosymbiont community established may vary with the thermal environment. Potential competitive advantages under different environmental conditions are also likely to underlie shuffling responses documented in some adult corals that host multiple *Symbiodinium* types [6], [9], [15]. While this may be an important acclimatization mechanism in adult corals with established symbioses, it is not known whether similar competitive processes occur within coral juveniles during initial *Symbiodinium* uptake.

Given that temperature and light are the main triggers for the breakup of the coral-*Symbiodinium* association (bleaching), it is critical to improve our understanding of how these factors might affect the establishment of the symbiosis and the type of *Symbiodinium* acquired. Such knowledge will enable predictions about how these vital symbiotic relationships might change in response to warming oceans. This study uses experimental manipulations to examine whether light and temperature influence the densities of *Symbiodinium* established in juveniles of *Acropora tenuis* and *A. millepora*, and also if these factors affect the type of *Symbiodinium* acquired. *Symbiodinium* preference was evaluated by comparing relative uptake of a type D, typically characterized as heat tolerant [8], [14], [33], versus type C1 under differing combinations of temperature and light. Specifically, we tested: (1) if coral juveniles are able to acquire symbionts equally well under different combinations of temperature and light, and (2) how the proportional abundance of two *Symbiodinium* types taken up by coral juveniles differs under differing temperature and light treatments.

## Materials and Methods

### Experimental corals, *Symbiodinium* inoculation, and genetic identification

Juvenile corals of *A. millepora* and *Acropora tenuis* were raised and settled on terracotta tiles after the coral spawning events of 2005 and 2007, respectively, at Magnetic Island (19°.10′S, 146°.50′E), an inshore island in the central Great Barrier Reef. Rearing and settling of coral larvae followed methods described in Puill-Stephan et al. [34]. *Symbiodinium* ITS1 types C1 and D, freshly isolated from adult colonies of *A. tenuis* and *A. millepora* from Magnetic Island, respectively, were used to inoculate juveniles of each species that had been raised in 0.5 µm filtered seawater and thus were *Symbiodinium-*free until provided with inoculants. *Symbiodinium* types were chosen because both have been detected in juveniles of the two coral species at Magnetic Island [35]. *Symbiodinium* cells were isolated by carefully blasting tissue from coral fragments using a compressed air gun inside plastic bags with a small volume (10–15 mL) of filtered sea water (FSW, filtered down to 1 µm). To obtain algal cells with as little host tissue as possible, the tissue slurry was filtered through 60 µm nylon mesh, homogenized for 1 minute (bench top homogenizer), and centrifuged (3000 rpm for 3 minutes) to concentrate the algal cells. Seawater with host tissue debris was decanted before re-suspending algal pellets in FSW. This process was repeated at least three times or until very little host tissue debris could be observed microscopically in re-suspended algal solutions. Mean cell densities were calculated from 6 replicate haemocytometer counts (six single field counts of six different droplets from each homogenized sample). Both types of *Symbiodinium* were offered simultaneously to coral juveniles in equal densities and volumes (see below). All necessary permits to conduct this research at Magnetic and Orpheus Islands were obtained from the Great Barrier Reef Marine Park Authority (permits G08/27313.1 and G05/13657.1 to B. Willis and D. Abrego). Ethics approval was not required for any of the research described in this study.

### Experimental design

Tiles with two week-old coral juveniles were placed in three temperature (28°C, 30°C, 31°C) and two light treatments (Low light: 180 µmol photons m^−2^ s^−1^; and High light: 390 µmol photons m^−2^ s^−1^) in temperature-controlled rooms at Orpheus Island Research Station (OIRS). Light levels were chosen to reflect the high turbidity environment of Magnetic Island, where the parental colonies (and sources of *Symbiodinium*) were collected. Light levels on the reef at Magnetic Island can range from 50–300 µmol photons m^−2^ s^−1^ [36], [37]. The photoperiod was 10 hrs light: 14 hrs dark. Within each of the six temperature by light treatments, tiles with corals were haphazardly assigned to four replicate containers supplied with flow-through filtered seawater (1 µm). The number of settled juveniles on each tile varied but approximately the same number of juveniles was allocated to each treatment. The volume of water in the containers was 7 L and flow rate into the containers was approximately 0.5 L minute^−1^. A small airstone in each container provided a constant stream of micro-bubbles throughout the experiment. Corals were maintained in these temperature by light treatments for 20 (*A. tenuis*) or 30 days (*A. millepora*). This difference was due to operational constraints at OIRS, which could not accommodate a 30 day experiment when juveniles of *A. tenuis* were available.

To explore the differential uptake of *Symbiodinium* in coral juveniles, types C1 and D were added simultaneously to containers every day during the first half of each experiment. The density of symbionts added to containers ranged between 2.3–6.7×10^3^ cells mL^−1^. The density varied because symbionts were freshly isolated prior to each inoculation. However, C1 and D symbiont densities were equalized and added in the same volumes to each container. Thus, while the total number of cells added to the containers was not the same at every inoculation, the proportion of C1 and D cells going into each container was always 1∶1 and the number of cells going into each container was the same at every inoculation. Water flow through the containers was stopped immediately before adding *Symbiodinium* and containers were maintained as static cultures for 16–18 hours. After this incubation period, flow was restored to flush containers with new seawater for approximately 6 hours before inoculations were repeated. Corals were maintained in filtered seawater (1 µm) throughout the experimental period. No *Symbiodinium* cells were added after the half-way point in each experiment in order to explore symbiont dynamics during the establishment phase, when both *Symbiodinium* types were present.

### Effects of temperature and light on *Symbiodinium* uptake

**Figure 1:**
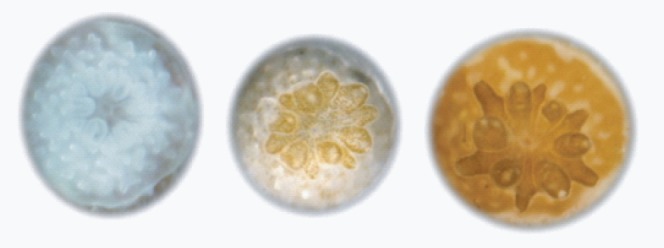
Visual assessment of *Symbiodinium* uptake. Juveniles were scored in two categories (white or pigmented) according to pigmentation levels. The specimen on the left is a typical “white” juvenile while the two on the right represent a range of “pigmented” juveniles.

To assess the impact of temperature and light on the combined uptake of the two *Symbiodinium* types, the number of juveniles in each treatment was counted on the last day of *Symbiodinium* inoculation (day 10 for *A. tenuis* and day 15 for *A. millepora*) and the level of uptake assessed visually. Each juvenile was assigned to one of two categories (more categories would have introduced subjective bias); juveniles were scored as white when no pigmentation was visible under a dissecting microscope (see Fig. 1), or pigmented when the oral disc, tentacles or walls of juvenile polyps exhibited pigmentation (Fig. 1, see Table 1 for sample sizes). Qualitative visual scoring was repeated on the last day of the experiment (day 20 for *A. tenuis* and 30 for *A. millepora*), when well over 100 juveniles remained in almost all of the 24 containers. Counts for white and pigmented juveniles were used to calculate the proportion of juveniles with visible pigmentation in each container (number of pigmented juveniles divided by the total number of juveniles in the container) as a rough measure of uptake efficiency, hereafter referred to as pigmentation ratio. In addition, the relative survival of juveniles in each temperature by light treatment was calculated based on the number of surviving juveniles at the end of the experiment relative to the number of juveniles at the end of the inoculation phase (mid-point of the experiment). The number of juveniles sub-sampled between the mid- and end-point censuses was subtracted from the total to avoid underestimating relative survival.

**Table 1:** Summary of total number of juveniles counted at each temperature by light treatment during the mid-experiment census (mid) and for the census at the end of the experiment (end).

	High light treatment		Low light treatment	
Temperature	n (mid)	n (end)	n (mid)	n (end)
***Acropora tenuis***				
28°C	2350	1316	2943	1844
29°C	2394	1279	2294	1050
31°C	2682	1064	2146	663
***A. millepora***				
28°C	354	59	319	121
30°C	147	7	235	73
31°C	180	40	271	84

To quantify symbiont uptake, samples of *A. tenuis* were collected and the number of *Symbiodinium* cells within each juvenile counted. Twelve samples were collected from each treatment (3 for each replicate container) on days 2, 10, and 20 of the experiment. Juveniles were randomly selected and carefully scraped off tiles using a scalpel blade and fixed in 10% formaldehyde in FSW. Samples were decalcified overnight in 5% formic acid. The acid was removed and the sample was homogenized in 300 µL of milli Q water using a small dispersion tool on a bench top homogenizer at maximum speed. Prior to homogenization, 30 µL of Alcian Blue dye were added to the sample to facilitate cell counting by pigmenting cell walls. Pigmented samples were kept on the bench for 15 minutes before mixing well with a micropipette and loading 10 µL of mixtures into a haemocytometer chamber. Each sample was counted 10 times. The number of cells was normalized to the number of polyps in each sample (recorded when the samples were scraped off the tiles).

### Effect of temperature and light on the type of *Symbiodinium* acquired and maintained by coral juveniles

To quantify whether the establishment of *Symbiodinium* types C1 and D occurred in equal ratios in juveniles in each of the six temperature by light treatments, coral juveniles were sub-sampled every other day during the experiment. At each sampling, 10 (*A. millepora*) or 20 (*A. tenuis*) juveniles were randomly sampled from each treatment and fixed in absolute ethanol. Higher initial numbers of *A. tenuis* juveniles enabled the higher sample numbers for this species. Extraction of total (coral and algal) DNA from each sub-sample followed a cetyltrimethyl ammonium bromide (CTAB)-based protocol modified from Hoarau *et al*. [38]. Briefly, each sample was placed in 250 µL of extraction buffer (2% CTAB, 0.1 M Tris (pH 9), 20 mM EDTA (pH 9), 1.4 M NaCl) and macerated inside a 1.5 mL micro-centrifuge tube using a disposable paper clip. The sample was incubated overnight at 60°C followed by addition of 250 µL of chloroform/isoamyl-alchohol (24∶1). After thorough mixing, the sample was centrifuged for 15 minutes at maximum speed in a bench top centrifuge. The aqueous phase was pipetted into a new tube and 250 µL of ice-cold 2-propanol was added followed by gentle mixing. The sample was incubated at −20°C for 20 minutes followed by centrifugation at maximum speed at room temperature for 20 minutes. The supernatant was discarded and the pellet washed with 150 µL of 70% ethanol followed by centrifugation for 5 minutes at maximum speed. The pellet was air-dried for 5 minutes and allowed to resuspend overnight in 400 µL of 0.01 M Tris (pH 9).

To quantify the relative abundance of *Symbiodinium* C1 versus *Symbiodinium* D established under different temperature×light treatments, two µL of DNA template were used for a real-time PCR assay, using C- and D-specific ITS1 primers developed by Ulstrup and van Oppen [39]. The real-time PCR reaction, containing 10 µL Sybr-Green Super Mix (Invitrogen), 2 µL ITS1 universal forward primer (180 nM), 2 µL ITS1 C or D reverse primer (180 nM), 4 µL of milli-Q water, and 2 µL of DNA template, was run on a Rotor Gene 3000 (Corbett Research). The reaction profile (following an initial heating step to activate the Taq polymerase as per manufacturer's recommendation) consisted of 40 two-step cycles of 15 s at 95°C and 30 s at 60°C. A melt curve was generated at the end of each run starting at 60°C and ramping to 95°C by increasing 0.5°C every five seconds (except for the first step, which was held for 45 seconds). Data acquisition took place during the 60°C step in each cycle, as well as during the melt curve period. The cycle-threshold (*C_T_*) was set to a fixed value for all runs to allow comparisons between runs. All reactions were run in duplicate. No-template controls and positive controls were included in every run. Data were collected using the Rotor Gene software (v 6.1). The relative abundance (cell ratios) of each *Symbiodinium* type was calculated by the 2-ΔΔ *C_T_* method, taking into account a difference in copy number between clades C and D of three (3 copies of D for every C), as described in Mieog *et al.* [40]. Cell ratios (D∶C ratios) were converted to proportions whereby 1 represented a *Symbiodinium* D only sample with no background of C and 0 represented a C sample with no background of D *Symbiodinium*.

### Data analysis

*Symbiodinium* cell counts and pigmentation ratios were log transformed and analyzed by three-factor ANOVA, with light (2 levels), day (2 levels for pigmentation ratio, 3 for cell counts), and temperature (3 levels) as fixed factors. Homogeneity of variance and normality were verified by Levene's test, spread vs. residual plots, and Q-Q plots. Pigmentation ratios for *A. millepora* juveniles were analyzed by Kruskal-Wallis test (temperature) and Mann-Whitney U test (light) as these data did not meet the assumptions of ANOVA after transformation. Relative survival was analyzed by two-way ANOVA with light (two levels) and temperature (3 levels) as fixed factors. Mann-Whitney U and Kruskal-Wallis tests were used for *A. millepora* juveniles as described above as these data did not meet assumptions of ANOVA. The effects of temperature and light on the D∶C cell ratios through time were analyzed by a two factor repeated measures ANOVA. Time (day) was treated as the within subject factor and temperature (three levels, fixed) and light (two levels, fixed) were treated as between subject factors. The assumption of sphericity was checked by Mauchly's test. All analyses were performed using SPSS software v. 16.0.

## Results

### Effects of temperature and light on the onset of symbiosis

**Figure 2:**
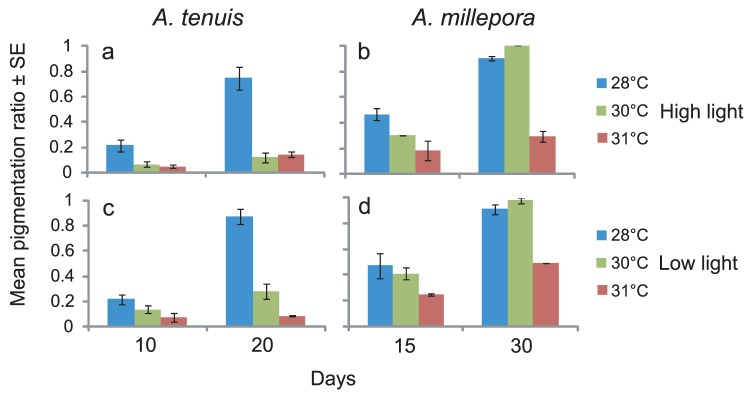
Pigmentation ratios (±1 SE) of coral juveniles kept at 28, 30, or 31°C and under high light (a,b; 390 µmol photons m^−2^ s^−1^) or low light (c,d; 180 µmol photons m^−2^ s^−1^) levels. Pigmentation ratios were calculated after 10 (*A. tenuis* a, c) or 15 (*A. millepora* b, d) days of exposure to *Symbiodinium*, and again after a further 10 or 15 days in filtered sea water (1 µm) without additional exposure to *Symbiodinium*. See Table 1 for sample sizes.

**Figure 3:**
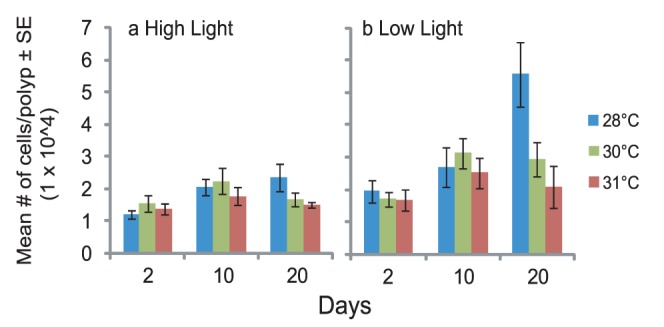
*Symbiodinium* cell counts in *A. tenuis* juveniles kept at 28, 30, or 31°C and under high light (a, 390 µmol photons m^−2^ s^−1^) or low light (b, 180 µmol photons m^−2^ s^−1^) levels. The number of cells was calculated from 10 replicate counts for each of 12 samples per treatment per day. Cell counts (±1 SE) were normalized to the number of polyps taken by each sample.

Qualitative visual assessments of coral juveniles indicated that elevated temperature had a strong negative effect on the uptake and establishment of *Symbiodinium* cells in both coral species. At the end of the inoculation phase (day 10), the proportion of pigmented juveniles differed significantly between the 28°C and 31°C treatments for *Acropora tenuis*, with up to four times more juveniles showing signs of *Symbiodinium* uptake at 28°C (Fig. 2a, c, ANOVA, p<0.001, Table 2). This difference was magnified further by the end of the experiment, when the proportion of juveniles with pigmentation was ten-fold greater at 28°C than at 31°C (Fig. 2c). The impact of light on the proportion of pigmented juveniles was only evident at 30°C, with *A. tenuis* juveniles in the low light treatment having significantly higher pigmentation ratios than those in the high light treatment (Fig. 2a, c, ANOVA, p<0.037, Table 2). These qualitative assessments were confirmed by a steady increase in cell counts over time in juveniles of *A. tenuis* at 28°C, resulting in significantly higher cell counts than found in the two higher temperature treatments (Fig. 3, ANOVA, p = 0.046, Table 3). Conversely, there was a declining trend in mean cell counts in the two higher temperature treatments between day ten and the end of the experiment (Fig. 3a, b). Furthermore, cell counts in *A. tenuis* differed significantly between light treatments, as well as among temperature treatments, with juveniles in the low light treatment having significantly higher cell counts than those in the high light treatment (Fig. 3, ANOVA, p<0.001, Table 3). This was in contrast to visual assessments of pigmentation, which did not detect any effect of light treatment on either coral species. For *A. millepora,* the proportion of pigmented juveniles, both at the end of the inoculation phase (day 15) and the end of the experiment, were up to three times greater at 28°C and 30°C than at 31°C (Figs. 2b, d).

**Table 2:** ANOVA results comparing the pigmentation ratio in *A. tenuis* juveniles exposed to three temperatures (28, 30, or 31°C) by two light levels (390 µmol photons m^−2^ s^−1^ or 180 µmol photons m^−2^ s^−1^) over a period of 20 days.

Factor	Degrees of freedom	F	Sig.	Tukey's
Day	1	39.975	<0.001	
Light	1	3.595	0.068	
Temp	2	1.872	<0.001	28>30>31
Day×Light	2	0.305	0.585	
Day×Temp	2	2.731	0.083	
Light×Temp	2	3.707	.037	
Light×Temp×Day	2	0.762	0.476	

Samples were taken on days 10 and 20. Fixed factors were Day, Light, and Temperature. Significant differences were further explored by Tukey's test.

**Table 3:** ANOVA results comparing the number of cells per polyp found in *A. tenuis* juveniles exposed to three temperatures (28, 30, or 31°C) by two light levels (390 µmol photons m^−2^ s^−1^ or 180 µmol photons m^−2^ s^−1^) over a period of 20 days.

Factor	Degrees of freedom	F	Sig.	Tukey's
Day	2	12.243	<0.001	2<10,20
Light	1	15.514	<0.001	
Temp	2	3.139	0.046	28>31
Day×Light	2	0.973	0.380	
Day×Temp	4	2.178	0.073	
Light×Temp	2	0.645	0.526	
Light×Temp×Day	4	0.787	0.535	

Samples were taken on days 2, 10 and 20. Fixed factors were Day, Light, and Temperature. Significant differences were further explored by Tukey's test.

**Figure 4:**
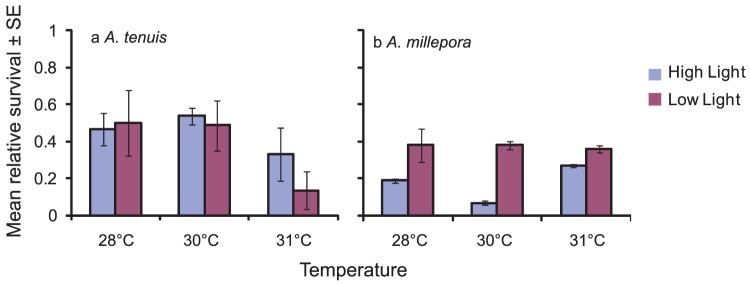
Relative survival (±1 SE) of juveniles in the 28, 30, or 31°C treatments and under high light (390 µmol photons m^−2^ s^−1^) or low light (180 µmol photons m^−2^ s^−1^) levels for: (a) *Acropora tenuis* after 20 days, and (b) *A. millepora* after 30 days. See Table 1 for sample sizes.

For juveniles of *A. tenuis* maintained in low light conditions, mean survival was up to four times higher at 28°C than at 31°C, suggesting an impact of temperature on survival at low light levels, although the trend was not statistically significant (ANOVA, F = 2.712, p = 0.099; Fig. 4a). Conversely, within each temperature treatment, the proportion of juveniles surviving did not differ between low and high light levels (ANOVA, F = 0.446, p = 0.514; Fig. 4a), suggesting that light had minimal impact on juvenile survival for *A. tenuis*. In contrast, light had a significant impact on the proportion of juveniles surviving for *A. millepora* at each temperature. A significantly greater proportion of juveniles survived in the low light treatment at each of the three temperatures (Fig. 4b. Mann-Whitney U test, U = 35.0, p = 0.006). Overall, the proportion of juveniles surviving did not differ among temperature treatments under low light, whereas under high light, there was a marked drop in survival in the intermediate (30°C) temperature treatment compared to the low and high temperature treatments.

### Effects of temperature and light on type of symbiont acquired and maintained

**Figure 5:**
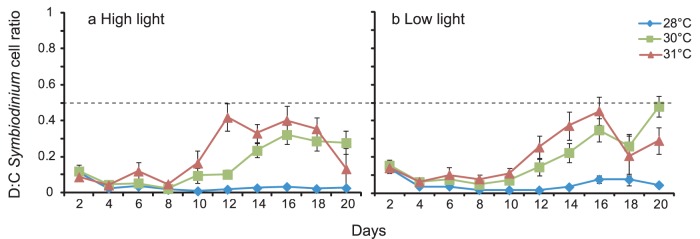
Change in *Symbiodinium* D∶C cell ratios (±1 SE) over time in *Acropora tenuis* juveniles at 28, 30, or 31°C in: (a) high light (390 µmol photons m^−2^ s^−1^), or (b) low light levels (180 µmol photons m^−2^ s^−1^). Dotted line represents equal proportions of *Symbiodinium* types D and C cells within the juveniles. Ratios closer to 1 are dominated by type D; ratios closer to 0 are dominated by type C. N = 20 per data point.

**Figure 6:**
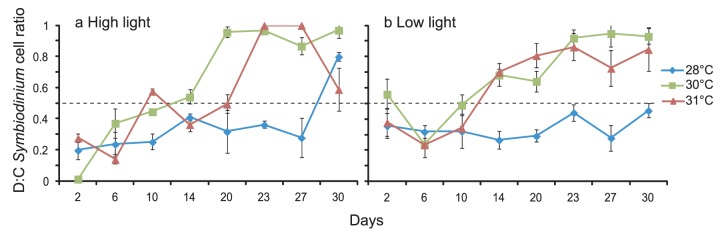
Change in *Symbiodinium* D∶C cell ratios (±1 SE) over time in *Acropora millepora* juveniles at 28, 30, or 31°C in: (a) high light (390 µmol photons m^−2^ s^−1^), or (b) low light levels (180 µmol photons m^−2^ s^−1^). Dotted line represents equal proportions of *Symbiodinium* types D and C cells within the juveniles. Ratios closer to 1 are dominated by type D; ratios closer to 0 are dominated by type C. N = 10 per data point.

Elevated temperatures had the overall effect of significantly increasing the D∶C cell ratio in juveniles of both coral species by the end of the experimental period (Tables 4–5), regardless of the light level (Figs. 5a–b, 6a–b). For juveniles of *A. tenuis*, *Symbiodinium* communities in all treatments were initially dominated by type C1 (D∶C cell ratios <0.5, Figs. 5a–b). D∶C cell ratios decreased initially during the inoculation phase (first 10 days) and were very similar across all temperature and light treatments. However, once *Symbiodinium* inoculations ceased half way through the experimental period, D∶C cell ratios increased in juveniles at 30°C and 31°C but not at 28°C (Figs. 5a–b). This pattern was similar for juveniles of *A. tenuis* in both light levels until the end of the experiment, when the D∶C ratio in corals at 30°C reached levels more than two-fold higher than those at 31°C (although they remained below 0.5, Figs. 5a–b).

**Table 4:** Repeated measures ANOVA results comparing changes in D∶C cell ratios in *Acropora tenuis* juveniles kept at three temperatures (28, 30, or 31°C) by two light levels (390 µmol photons m^−2^ s^−1^ or 180 µmol photons m^−2^ s^−1^).

*Source of variation*	*Sum of squares*	*Degrees of freedom*	*Mean square*	*F*	*Sig.*	*Tukey's*
Within Subjects analysis						
Day	0.497	9	0.055	3.241	0.001	
Day×Light	0.380	9	0.042	2.478	0.012	
Day×Temp	0.987	18	0.055	3.216	<0.001	
Day×Light×Temp	0.317	18	0.018	1.034	0.427	
Between Subject analysis						
Light	0.024	1	0.024	1.229	0.286	
Temp	0.758	2	0.379	19.020	<0.001	28<30, 31
Light×Temp	0.084	2	0.042	2.118	0.157	

Within-subjects factor Day was 2, 4, 6, 8, 10, 12, 14, 16, 18, and 20.

**Table 5:** Repeated measures ANOVA results comparing changes in D∶C cell ratios in *A. millepora* juveniles kept at three temperatures (28, 30, or 31°C) by two light levels (390 µmol photons m^−2^ s^−1^ or 180 µmol photons m^−2^ s^−1^).

Within Subject analysis						
Day	4.874	7	0.696	33.621	<0.001	
Day×Light	0.400	7	0.057	2.757	0.013	
Day×Temp	1.613	14	0.115	5.565	<0.001	
Day×Light×Temp	1.149	14	0.082	3.964	<0.001	
Between Subject analysis						
Light	0.020	1	0.020	1.458	0.253	
Temp	2.412	2	1.206	85.858	<0.001	28<30, 31
Light×Temp	0.041	2	0.020	1.456	0.275	

Within-subjects factor Day was 2, 6, 10, 14, 20, 23, 27, and 30.

Initially, *Symbiodinium* communities in juveniles of *A. millepora* were dominated by type C1 at high light levels for all temperatures (Fig. 6a). However, unlike *A. tenuis*, D∶C cell ratios increased during the inoculation phase, reaching approximately equal densities by the end of this period (day 15, Fig. 6a). D∶C cell ratios in the higher temperature treatments continued to increase after *Symbiodinium* inoculations ceased, but the D∶C cell ratio in juveniles at 28°C remained close to 0.5 until the last sampling point, when it reached 0.8, indicating a D-dominated symbiosis (Fig. 6a). For juveniles at the low light level, the D∶C cell ratios also increased over time in the 30°C and 31°C treatments but remained at approximately equal densities in the control treatment (28°C, Fig. 6b).

## Discussion

**Figure 7:**
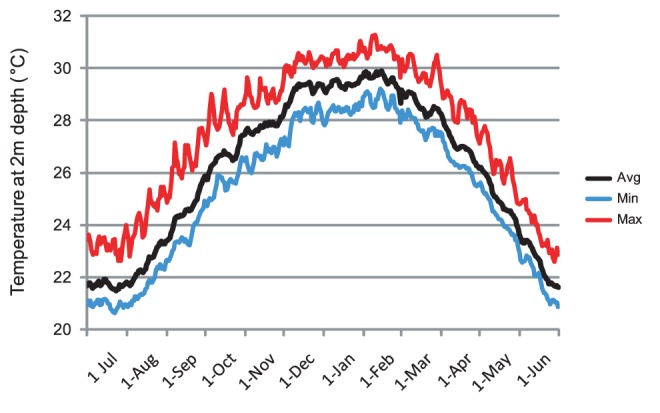
Long term sea temperature at 2 m depth in Geoffrey Bay, Magnetic Island. Data was collected over 15 years (1996–2011) by loggers maintained by the Australian Institute of Marine Science (Channel ID 1820, data available from http://data.aims.gov.au/gbroosdata/services/rss/channel/923/150).

This study highlights the importance of temperature and light for the uptake and establishment of *Symbiodinium* symbioses in coral juveniles. Our results show that elevated temperatures have a significant impact on the establishment of the initial symbiosis in coral juveniles, similar to the responses found for coral larvae to elevated temperatures [29]–[31]. The low level of *Symbiodinium* uptake at the two higher temperatures, in comparison to the 10-fold higher pigmentation ratio and up to 2.6 fold increase in *Symbiodinium* cells in juveniles kept at 28°C, has important implications for coral persistence given projected increases in sea surface temperatures associated with climate change [28]. First, acquisition of *Symbiodinium* by newly settled juveniles of broadcast spawning corals on the Great Barrier Reef occurs during late spring and early summer [41], when seawater temperatures are typically increasing. At Magnetic Island, corals generally spawn in October or November when seawater temperatures typically reach 28°C and continue to warm through February (Fig. 7). Elevated temperatures may compromise the formation of the symbiosis by inducing oxidative stress in both host and algal cells. High levels of superoxide dismutase (SOD) and malondialdehyde (MDA) were detected in coral larvae exposed to 32°C [30]. Moreover, both SOD and MDA levels were more than double in larvae with algal cells than in *Symbiodinium*-free larvae, suggesting that algal cells may become a liability to the host at high temperatures by increasing oxidative stress and ultimately higher mortality [30]. Lower survival of symbiotic compared to non-symbiotic larvae at high temperatures has also been documented by Schnitzler et al [29]. However, low rates of infection due to elevated temperatures may result in low survival of recruits and/or slow growth of juveniles as a consequence of diminished transfer of photosynthates to the coral host [42]. Although patterns of juvenile survival over the time span covered here were not significantly different among temperature treatments, it is unlikely that uninfected juveniles would have survived for longer periods of time, given the fundamental role of algal endosymbiosis in coral survival [1].

Counts of *Symbiodinium* cells in juveniles of *A. tenuis* clearly show that light has a significant impact on the uptake of algal symbionts (Fig. 3). The higher number of symbionts in the low light treatment across all temperatures may be explained by lower levels of oxidative stress on these cells compared to those at high light levels. Under normal conditions, *Symbiodinium* cells are capable of protecting themselves from oxidative damage by non-photochemical quenching [43]. However, these mechanisms can be overwhelmed by high light or high temperature stress, which may explain why *A. tenuis* juveniles at 31°C in the high light treatment had the lowest *Symbiodinium* cell counts. The role of light was also evident by the significant interaction between light and temperature in the qualitative visual assessments of *Symbiodinium* uptake, which was driven by higher pigmentation ratios in juveniles exposed to low light at 30°C than those in high light. Light had no effect on symbiont densities in a previous four-day study using coral larvae maintained in either ambient light levels or virtual darkness [24]. However, these results may have reflected the short duration of the experiment, as a possible lag phase in *Symbiodinium* population growth immediately following inoculation [44], may have prevented the detection of light effects in that study.

Overall patterns of uptake were similar for both coral species but based on qualitative assays, uptake was greater in *A. millepora* juveniles. Two factors could have influenced these results. First, the duration of the experiment for *A. millepora* was longer. Hence, corals had a longer period of exposure to symbionts before our observations took place. Second, the number of *A. millepora* juveniles available for the experiment was lower. Since the quantity of *Symbiodinium* cells added to both coral species was similar and assuming that uptake occurs upon contact with a juvenile, the number of cells available to potentially infect *A. millepora* juveniles was greater and may have contributed to greater uptake. It is worth noting that the proportion of C1 and D cells added to both coral species was equal, so the probability of exposure to either type was the same, even if the overall probability of coming into contact with a symbiont was higher for *A. millepora* juveniles.

Elevated temperatures favoured uptake of a thermally tolerant *Symbiodinium* type by juveniles of both coral species. Relative to *Symbiodinium* C1, *Symbiodinium* D used in the present study has been shown to confer a small increase in the thermal tolerance of adult colonies [15] and juveniles [45] of *A. millepora,* but not of juveniles of *A. tenuis* [10]. In the present study, increases in D∶C cell ratios over time in juveniles of both species are consistent with the view that type D is better at infecting and/or replicating within corals at high temperatures. It is important to recognize that while the patterns observed in our study may be the result of competitive interactions between C1 and D symbionts, a similar outcome could result in the absence of direct competition. Type D symbionts may simply thrive in warmer temperatures while C1 do not, thus type D symbionts could colonize space not occupied by C1 symbionts without actually displacing them. Although patterns of greater uptake of type D detected in our study are at odds with results of an earlier study showing that the thermal tolerance of *A. tenuis* juveniles is not enhanced by associating with clade D *Symbiodinium* compared to C1 [10], they are consistent with field observations of *Symbiodinium* type D uptake by coral juveniles (regardless of the type found in adult colonies) a few weeks after annual spawning events, when water temperatures are approaching their highest levels [27]. However, the benefits of associating with *Symbiodinium* D to the coral host remain to be fully explored given that juveniles at high temperatures had low pigmentation ratios and *Symbiodinium* cell numbers and may therefore be photosynthate-limited. This likely impedes rapid growth of juvenile colonies and makes them more prone to mortality [25], [46], even if these corals are able to feed heterotrophically to compensate for possible low photosynthate inputs [47].

Species-specific differences in the uptake of *Symbiodinium* documented in this study indicate the potential existence of host effects in the early stages of establishment of symbioses. These differences were particularly evident at the ambient control temperature, where high *Symbiodinium* cell numbers and pigmentation levels indicated successfully established symbioses. In *A. tenuis* juveniles, the association was almost entirely C-dominated by the end of the experiment (D∶C ratio <0.02), as is typical of *A. tenuis* adults at Magnetic Island (i.e. type C1 is homologous). In contrast, in *A. millepora* juveniles at the control temperature, the symbiosis was approximately equal in proportion (D∶C ratio of 0.45 at low light), or dominated by type D *Symbiodinium* (D∶C ratio of 0.8 at high light). Furthermore, juveniles at 30°C, which also had high pigmentation ratios, were almost completely dominated by type D *Symbiodinium* (D∶C ratio >0.93). This is the type found in *A. millepora* adults at Magnetic Island. Thus, the dominance of the homologous *Symbiodinium* type in both coral species at the control temperature shows that host effects play a role in the early establishment of the symbiosis. However, elevated temperatures may disrupt this interaction. *A. tenuis* juveniles at high temperatures had a higher proportion of type D *Symbiodinium* than conspecifics at 28°C. On the reef, uptake of *Symbiodinium* by *A. tenuis* juveniles is dominated by type D [27]. Initial uptake on the reef occurs in early summer, when sea-surface temperatures are rising towards maximum levels (see Fig. 7). Hence, high temperatures in the field may inhibit mechanisms by which host effects play a role in the uptake of homologous symbionts and result in non-specific uptake.

Our study provides insights into how different symbionts take up residence in newly settled corals. Corals are able to acquire *Symbiodinium* as early as the larval stage [22], [48], which means that larvae on the reef may be exposed to symbionts before settlement. It is not known whether recruits in the field lack symbionts before settlement or the extent to which corals that have acquired symbionts as larvae are able to do so after settlement. Several studies provide evidence that initial uptake of symbionts by coral juveniles is a dynamic process that can extend for weeks to years before a stable association is formed [23], [25]–[27], [35]. Moreover, findings of higher densities of *Symbiodinium* and *Symbiodinium*-like algae in reef sediments than in the water column [32], [49], [50], along with periodic motile phases observed for *Symbiodinium* in culture, suggest that encounters with settled juveniles on the reef are likely. It is also worth noting that coral larvae do not require contact with *Symbiodinium* in order to settle and metamorphose [51], [52].

Inherent physiological differences between the two *Symbiodinium* types may provide a competitive edge to type D at elevated temperatures or may allow this symbiont type to quickly occupy available space in the absence of direct competition with other symbiont types. However, inherent physiological differences alone cannot explain the host-specific patterns in uptake and establishment detected in this study showing that host effects in closely related coral species have significant impacts in early symbiont uptake and establishment of the symbiosis.
